# The accumulation of elements in plants growing spontaneously on small heaps left by the historical Zn-Pb ore mining

**DOI:** 10.1007/s11356-015-5859-7

**Published:** 2015-12-04

**Authors:** Anna M. Stefanowicz, Małgorzata Stanek, Marcin W. Woch, Paweł Kapusta

**Affiliations:** W. Szafer Institute of Botany, Polish Academy of Sciences, Lubicz 46, 31-512 Kraków, Poland; Institute of Biology, Pedagogical University of Kraków, Podchorążych 2, 31-054 Kraków, Poland

**Keywords:** Historical mining, Heavy metal accumulation, Plant tissues, Bioconcentration factor, Translocation factor

## Abstract

The study evaluated the levels of nine metals, namely Ca, Cd, Fe, K, Mg, Mn, Pb, Tl, and Zn, in soils and tissues of ten plant species growing spontaneously on heaps left by historical mining for Zn-Pb ores. The concentrations of Cd, Pb, Tl, and Zn in heap soils were much higher than in control soils. Plants growing on heaps accumulated excessive amounts of these elements in tissues, on average 1.3–52 mg Cd kg^-1^, 9.4–254 mg Pb kg^-1^, 0.06–23 mg Tl kg^-1^ and 134–1479 mg Zn kg^-1^ in comparison to 0.5-1.1 mg Cd kg^-1^, 2.1–11 mg Pb kg^-1^, 0.02–0.06 mg Tl kg^-1^, and 23–124 mg Zn kg^-1^ in control plants. The highest concentrations of Cd, Pb, and Zn were found in the roots of *Euphorbia cyparissias*, *Fragaria vesca*, and *Potentilla arenaria*, and Tl in *Plantago lanceolata*. Many species growing on heaps were enriched in K and Mg, and depleted in Ca, Fe, and Mn. The concentrations of all elements in plant tissues were dependent on species, organ (root vs. shoot), and species-organ interactions. Average concentrations of Ca, K, and Mg were generally higher in shoots than in roots or similar in the two organs, whereas Cd, Fe, Pb, Tl, and Zn were accumulated predominantly in the roots. Our results imply that heaps left by historical mining for Zn-Pb ores may pose a potential threat to the environment and human health.

**Electronic supplementary material:**

The online version of this article (doi:10.1007/s11356-015-5859-7) contains supplementary material, which is available to authorized users.

## Introduction

Contaminated sites left by historical mining and processing of metal ores, abandoned tens or hundreds of years ago, may be difficult to evaluate in terms of environmental risk, as they are often not recorded in written documents and not known to the local authorities (Eckel et al. 2001). Relics of the former metal industry, such as waste heaps, tend to disappear from the landscape, for example as a result of the denudation process. Even if the waste heaps can be visually detected in the field, their inventory is still a difficult task because the heaps are often small and scattered over a wide area (Aleksander-Kwaterczak and Ciszewski 2013; Stefanowicz et al. 2014). Unfortunately, the disappearance of traces of the ancient metal industry from the landscape is not followed by the loss of heavy metals from the environment. It is known that these metals may persist in high concentrations for decades, centuries, or even millennia not only at the contamination sources but also in adjacent agricultural soils, sediments, and surface waters, to which they are gradually transported (Adams et al. 2007; Grattan et al. 2003; Harrison et al. 2003; Pyatt et al. 2005; Stefanowicz et al. 2014; Tatsi and Turner 2014; Teršič et al. 2009).

Western Małopolska, located in southern Poland, is one of the oldest regions of mining and processing of metal ores in Central Europe. These activities have lasted there for centuries, leaving traces, such as old heaps of waste rock (Aleksander-Kwaterczak and Ciszewski 2013; Stefanowicz et al. 2014). The heaps are small, inconspicuous, and frequently masked by vegetation. They occur in high numbers over an extended area, singly or in groups, in the latter case forming a characteristic undulating surface. Recently, we analyzed soils developed on several dozen of non-forested heaps and found that they contain large amounts of heavy metals, in extreme cases: 520 mg Cd kg^-1^, 23,000 mg Pb kg^-1^, 50 mg Tl kg^-1^, and 70,400 mg Zn kg^-1^. These heaps are, therefore, “hot-spots” of metal contamination, which can affect neighboring agricultural land or vegetable gardens and pose a potential threat to human health (Stefanowicz et al. 2014).

Old heaps found in non-forested areas are covered with relatively dense vegetation (Woch et al. 2015). This vegetation consists predominantly of herbaceous species that commonly grow on unpolluted soils, but show a relatively high tolerance to heavy metals and are also able to colonize metalliferous soils (Woch et al. 2015). Although most herbaceous species growing on old heaps are small and do not produce great amount of biomass, they occur frequently, possibly exerting a meaningful influence on the metal fate in the ecosystem. Most of the plants that survive on metalliferous soils accumulate heavy metals in the roots and restrict their transport to the aerial parts, being potentially useful in phytostabilization, which focuses on immobilization of pollutants (Dahmani-Muller et al. 2000; Yoon et al. 2006). The opposite strategy is based on active transport of elements from roots to shoots, which leads to their accumulation in aboveground plant tissues (Pollard et al. 2014). In the case of heavy metals, such accumulation poses a risk of metal transfer to higher strata of the food chain (Peralta-Videa et al. 2009). Even if a given metal is not readily available in the soil, its uptake by an accumulator plant can make it available to soil organisms, insects, and grazers (Mertens et al. 2005). This problem was reported by Pyatt et al. (2000). who found that both plants and herbivores, including goats, living in a polluted area of ancient metal mining in Jordan, contained elevated heavy metal concentrations in their tissues.

The aim of this research was to analyze the concentrations of nine metals, namely Ca, Cd, Fe, K, Mg, Mn, Pb, Tl, and Zn, in shoots and roots of ten species of herbaceous plants growing spontaneously on the heaps of waste rock left by historical Zn-Pb ore mining. These concentrations were compared with the corresponding concentrations of metals in the plant from control sites and referred to metal contents in the soil. Plant/soil and shoot/root ratios were calculated to determine the strategy of particular plant species regarding metal management, the potential risk of heavy metal diffusion in the food web, and the usefulness of selected plant species in phytoremediation of heavy metal-contaminated lands.

## Materials and methods

### Study area and sampling

**Fig. 1:**
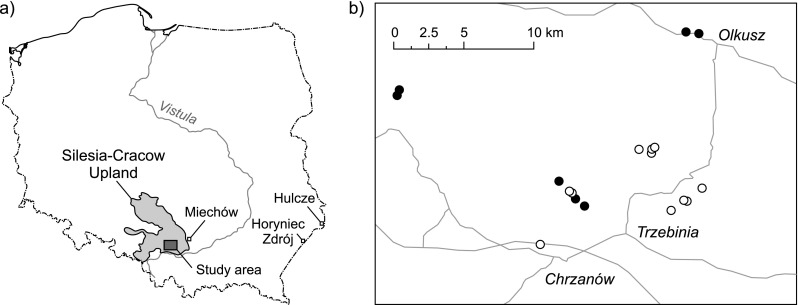
Location of (**a**) the study area and control sites and (**b**) heaps, from which ten (*black circles*) or five (*white circles*) plant species were collected. Major cities and main roads are also indicated

The location of the study area and waste heaps is presented in Fig. 1. The area was described in terms of geology and the history of Zn-Pb ore mining in Stefanowicz et al. (2014). The heaps were on average 0.5–2 m high and from several to tens m in diameter. They were covered by calcareous grasslands of *Carlino acaulis*-*Brometum erecti* association, the dominant variant of which was loose heavy metal grassland with *Festuca ovina* as the main component (Woch et al. 2015).

Soil and plant (shoots and roots) samples were collected in summer 2012. Ten herbaceous species were selected for the study: *Achillea collina* Becker ex Rchb. (Asteraceae), *Carex hirta* L. (Cyperaceae), *Euphorbia cyparissias* L. (Euphorbiaceae), *Fragaria vesca* L. (Rosaceae), *Hieracium pilosella* L. (Asteraceae), *Leontodon hispidus* L. (Asteraceae), *Plantago lanceolata* L. (Plantaginaceae), *Potentilla arenaria* Borkh. (Rosaceae), *Rumex acetosa* L. (Polygonaceae), and *Scabiosa ochroleuca* L. (Dipsacaceae). These species were chosen because they commonly occur on both contaminated and uncontaminated soils, and, with the exception of *P. lanceolata*, have been rarely studied in respect of metal accumulation (Dimitrova and Yurukova 2005; Nadgórska-Socha et al. 2013; Orłowska et al. 2002; Szarek-Łukaszewska and Niklińska 2002; Wójcik et al. 2014). All plant species were collected from seven heaps (one set of species per heap; Fig. 1). This sampling scheme allowed to compare tissue metal levels between species that grow under the same soil conditions, avoiding pseudoreplication. Samples of five species, i.e., *C. hirta*, *F. vesca*, *H. pilosella*, *P. arenaria*, *P. lanceolata*, were collected from 11 additional heaps to be able to study the relationships between soil and plant metal concentrations (*N* = 18; Fig. 1). Three topsoil (0–15 cm) subsamples were collected at each site following careful removal of the organic (O) horizon; the three subsamples were bulked into one composite sample. Five control sites were established near Horyniec Zdrój and Hulcze in south-eastern Poland and near Miechów in southern Poland, similar in geology to the main study area, but remote from anthropogenic sources of pollution (Fig. 1; Stefanowicz et al. 2014). Soil samples were collected from each control location as described above (*N* = 5). We were not able to find the whole set of plant species at all control sites, thus the control plants were collected from one to three sites (depending on the species). A single species sample from each heap and each control site consisted of three subsamples (specimens).

### Laboratory work

Soil samples were sieved (2-mm mesh) and dried at 105 °C. Soil texture was determined through a combination of sieving and sedimentation, and soil pH was tested with a pH-meter (Hach HQ40d) after extraction with water at a 1:5 (*w*:*v*) ratio. Organic carbon was assessed using a dry combustion technique with a Leco RC-612. Total nitrogen was measured using the Kjeldahl method; soil was digested in H_2_SO_4_ with Kjeltabs (K_2_SO_4_ + CuSO_4_ · 5H_2_O; Foss Tecator Digestor Auto) followed by distillation on the Foss Tecator Kjeltec 2300 Analyzer Unit. To determine the concentration of the different forms of metals in soil, four soil extraction methods were applied. Total Ca, Cd, Fe, K, Mg, Mn, Pb, Tl, and Zn were extracted by digestion of soil with hot concentrated HClO_4_ (Foss Tecator Digestor Auto). Mobile metals were extracted by shaking the soil samples for 1 h with 0.05 M EDTA (Cd, Pb, Tl, Zn), 0.1 M BaCl_2_ at pH 7.0 (Ca, Cd, Fe, K, Mg, Mn, Pb, Tl, Zn), and deionized water (Cd, Pb, Tl, Zn). Metal contents in soil extracts were analyzed with flame or graphite furnace (water-extractable metals) atomic absorption spectrometry (Varian AA220FS, GTA 110). Certified reference materials were used to estimate the quality of the metal analyses: CRM048-050 (RTC; total), BCR-483 (IRMM; EDTA-extractable), and ISE-912 (WEPAL; BaCl_2_-extractable); for EDTA and BaCl_2_, certified or indicative values were given only for selected metals. The recovery values ranged from 94 to 102 % for total metal contents, from 91 to 98 % for EDTA-extractable metals and from 93 to 97 % for BaCl_2_-extractable metals.

The collected plants were deprived of brown dead leaves and washed carefully in running tap water, followed by double distilled and deionized water. The plants were divided into shoots and roots and dried at 80 °C for 48 h. To analyze the elemental composition, the plant material was ground and digested in a hot concentrated mixture of HNO_3_ and HClO_4_ (4:1; Foss Tecator Digestor Auto). The concentrations of Ca, Cd, Fe, K, Mg, Mn, Pb, Tl, and Zn were analyzed using flame or graphite furnace atomic absorption spectrometry (Varian AA280FS; Varian AA280Z, GTA 120). Certified reference materials, Oriental Basma Tobacco Leaves INCT-OBTL-5 (The Institute of Nuclear Chemistry and Technology) and moss *Pleurozium schreberi* M2 (The Finnish Forest Research Institute) were used to test the quality of metal analyses in plants. The recovery values for plant extracts ranged from 92 to 113 %.

### Calculations and statistical analyses

For all metals and plant species, bioconcentration (BCF) and translocation factors (TF) were calculated. BCF is the ratio of plant/soil metal concentrations, and TF is the ratio of shoot/root metal concentrations. Considering the fact that the total Cd, Pb, Tl, and Zn concentrations can be extremely high in the post-mining sites, and BCF based on them could underestimate the ability of plants to transfer metals from the soil to the roots, EDTA-extractable metal-based BCF values (BCF_EDTA_) were also calculated for these elements.

The BaCl_2_-extractable Fe, Pb, and Tl were neither included in statistical analyses nor presented in tables, as most of the values were below the detection limits. Prior to statistical analyses, all variables were transformed with a logarithmic or exponential function and expressed on a 0–1 scale. Student’s *t* test and non-parametric Mann-Whitney’s *U* test were used to compare heap and control soils in terms of their physicochemical properties. Split-plot ANOVA was conducted to test the effect of plant species, the effect of plant organ (shoot vs. root), and the effect of species-organ interaction on element accumulation. This was followed by contrast analysis to estimate the differences in metal concentrations between the shoots and roots of particular species (Bonferroni correction for multiple comparisons was applied). Pearson’s correlation coefficients were calculated to test the relationships between metal concentrations in the soil and plants. Statistical analyses were performed with Statistica 9 (StatSoft Inc.).

## Results

### Physicochemical properties of the soil

Total heavy metal concentrations in heap soils ranged from 8 to 337 mg Cd kg^-1^, from 196 to 23006 mg Pb kg^-1^, from 14 to 35 mg Tl kg^-1^ and from 1126 to 42496 mg Zn kg^-1^, and were much higher than the concentrations in control soils (Table 1). The concentrations of mobile, i.e., BaCl_2_-, EDTA-, or water-extractable Cd, Pb, and Zn, but not Tl, were also higher in the heap soils than in the control soils (Table 1). The variability of both total and available, i.e., BaCl_2_-, EDTA-, or water-extractable Cd, Pb and Zn, and H_2_O-extractable Tl, among the heap soils was high. The coefficients of variation (CV) calculated for these parameters varied from 74 to 164 %, indicating a steep soil pollution gradient. In contrast, total and EDTA-extractable Tl was much less variable, with CV slightly exceeding 20 %. The concentrations of Cd, Pb, and Zn positively correlated with each other (*p* < 0.05; correlation coefficients ranged from 0.50 to 0.96), but not with Tl (*p* > 0.05; correlation coefficients ranged from 0.04 to 0.46). Significant positive correlations were found also between different forms of Cd, Pb, and Zn (*p* < 0.05; correlation coefficients varied from 0.73 to 0.96). Heap soils were similar to each other in terms of certain physicochemical properties. They were all alkaline and classified mostly as sandy loam. CV for pH and the contents of sand, silt and clay, alkali and alkaline earth metals, as well as C and N were relatively low, ranging from 3 to 73 %. Heap soils contained more C, N, Fe, Mg, Mn, and silt particles and had lower pH than the control soils (Table 1).

**Table 1:** Physicochemical characteristics of the soil collected from old heaps and control sites

	Heap soil (*N* = 18)			Control soil (*N* = 5)		
	minimum	mean (SD)	maximum	minimum	mean (SD)	maximum
Sand (%)**	45	61 (10)	84	70	81 (10)	96
Silt (%) (**)	7	21 (7)	30	2	6 (3)	11
Clay (%)	7	17 (9)	38	2	13 (8)	23
pH**	7.5	8.0 (0.2)	8.2	7.9	8.6 (0.5)	9.1
Organic C (%)*	1.2	3.3 (1.4)	6.7	0.1	1.5 (2.0)	4.9
Total N (%)*	0.15	0.25 (0.07)	0.42	0.02	0.13 (0.15)	0.39
Total Ca (g kg^-1^)	33	134 (70)	254	1	83 (81)	180
Total Cd (mg kg^-1^) (***)	8	118 (102)	337	2	4 (1)	5
Total Fe (g kg^-1^)***	8	41 (26)	102	1	8 (4)	13
Total K (g kg^-1^)	0.5	2.8 (2.0)	9.1	0.3	2.2 (1.6)	4.0
Total Mg (g kg^-1^) (***)	8.6	43.7 (21.6)	76.9	0.2	2.5 (1.5)	3.8
Total Mn (mg kg^-1^)***	296	1168 (766)	3169	37	163 (107)	315
Total Pb (mg kg^-1^) (***)	196	3987 (5354)	23006	13	40 (22)	67
Total Tl (mg kg^-1^)***	14	25 (6)	35	1	10 (8)	17
Total Zn (mg kg^-1^) (***)	1126	18814 (14698)	42496	63	191 (170)	476
BaCl_2_-extractable Ca (g kg^-1^)	1.6	2.2 (0.4)	2.9	0.5	1.6 (1.1)	3.1
BaCl_2_-extractable Cd (mg kg^-1^) (**)	bdl	4.7 (4.9)	14.7	bdl	bdl	bdl
BaCl_2_-extractable K (mg kg^-1^)	43	88 (28)	132	23	100 (96)	256
BaCl_2_-extractable Mg (mg kg^-1^) (***)	92	405 (128)	589	11	29 (17)	54
BaCl_2_-extractable Mn (mg kg^-1^)**	0.05	0.62 (0.38)	1.46	0.06	0.18 (0.11)	0.33
BaCl_2_-extractable Zn (mg kg^-1^) (***)	0.2	25.4 (33.6)	130.0	bdl	0.1 (0.1)	0.2
EDTA-extractable Cd (mg kg^-1^) (***)	1.9	40.2 (38.7)	141.1	0.1	0.3 (0.2)	0.5
EDTA-extractable Pb (mg kg^-1^) (**)	50	1268 (2083)	9092	6	18 (21)	56
EDTA-extractable Tl (mg kg^-1^)	1.0	1.4 (0.3)	2.0	0.3	1.3 (0.7)	1.8
EDTA-extractable Zn (mg kg^-1^) (***)	68	1489 (1104)	3673	3	6 (3)	10
water-extractable Cd (μg kg^-1^)***	3	33 (43)	146	1	3 (1)	5
water-extractable Pb (μg kg^-1^)***	218	1319 (1558)	4609	bdl	41 (32)	83
water-extractable Tl (μg kg^-1^)^nd^	bdl	1.6 (2.2)	9.0	nd	nd	nd
water-extractable Zn (mg kg^-1^) (***)	1.1	7.5 (6.6)	27.8	0.2	0.4 (0.1)	0.5

*bdl* below the detection limit: 0.02 mg Cd l^-1^ for flame AAS, 0.3 μg Pb l^-1^ and 0.1 μg Tl l^-1^ for graphite furnace AAS; *nd* not determined

*Asterisks* indicate significant differences between the heap and control soil (Student’s *t* test: *p < 0.05, **p < 0.01, ***p < 0.001; Mann-Whitney’s *U* test: (*) *p* < 0.05, (**) *p* < 0.01, (***) *p* < 0.001

### Concentrations of elements in plants

**Fig. 2:**
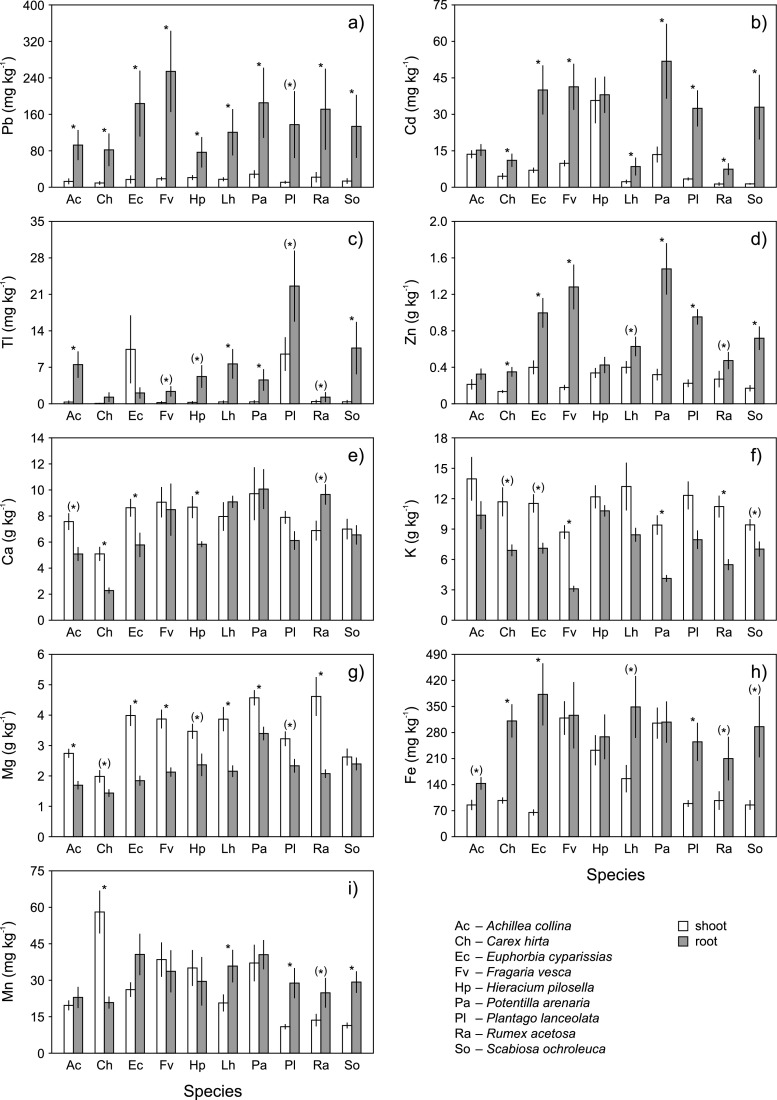
Comparison of element accumulation levels between plant species growing on the heaps left by historical Zn-Pb ore mining (means and standard errors, *N* = 7). Statistically significant differences in the element accumulation between plant organs are *asterisked*: *p < 0.005, Bonferroni correction applied; (*) 0.005 < *p* < 0.05

The concentrations of the pollution elements, i.e., Cd, Pb, Tl, and Zn, in the tissues of plants collected from heaps were high. They were on average 2.5–80 (Cd), 2.0–35 (Pb), 1.5–540 (Tl), and 2.7–20 (Zn) times higher than those determined for control plants (Table 2). The concentrations of heavy metals in heap plants were significantly (*p* < 0.05) affected by the plant species, plant organ (shoot vs. root) and the interaction of both factors. The pollution elements accumulated predominantly in roots; this was the most pronounced for Pb (Fig. 2a). The greatest amounts of Cd were found in the *P. arenaria* roots (on average 52 mg kg^-1^; Fig. 2b), Pb in the *F. vesca* roots (254 mg kg^-1^; Fig. 2a), Tl in the *P. lanceolata* roots (23 mg kg^-1^; Fig. 2c), and Zn in the *P. arenaria* roots (1479 mg kg^-1^; Fig. 2d). A different pattern was observed for the concentration of Tl in the *E. cyparissias* tissues: it tended to be higher in the shoots than in the roots (10 vs. 2.1 mg kg^-1^; Fig. 2c). The difference was not statistically significant probably due to extreme inter-heap variability of Tl shoot concentrations: they were low (<0.06 mg kg^-1^) in plants from four heaps and high (5.4–44 mg kg^-1^) in plants from three other locations. The accumulation of Cd, Pb, Tl, and Zn in plant tissues differed considerably between sites (Table S1). The highest inter-heap variability was detected for Tl, with CV often considerably exceeding 100 %. Very high variability was also found for Pb, but only in the case of its root concentrations.

**Table 2:** Ratios of average element concentrations in the heap plants to those in the control plants

		*A. collina*	*C. hirta*	*E. cyparissias*	*F. vesca*	*H. pilosella*	*L. hispidus*	*P. arenaria*	*P. lanceolata*	*R. acetosa*	*S. ochroleuca*
Ca	shoot	*0.9*	*0.8*	1.0	1.3	*0.9*	*0.9*	1.1	1.3	1.0	1.0
	root	*0.6*	*0.5*	*0.8*	*0.6*	*0.4*	*0.8*	7.3	*0.8*	5.3	*0.5*
Cd	shoot	28	5.9	14	9.1	37	3.2	9.6	6.0	2.5	2.9
	root	21	9.6	80	29	31	15	43	37	6.5	46
Fe	shoot	*0.5*	*0.9*	*0.5*	1.9	1.1	*0.8*	2.3	*0.7*	*0.9*	*0.8*
	root	*0.3*	*0.8*	*0.8*	*0.6*	*0.7*	*0.6*	*0.9*	*0.8*	*0.8*	*0.7*
K	shoot	1.4	1.4	1.6	1.9	1.3	1.3	1.1	1.5	1.3	1.3
	root	*0.8*	*0.9*	1.1	1.1	1.0	*0.9*	1.0	1.1	1.0	*0.9*
Mg	shoot	1.8	1.7	2.2	2.9	2.5	1.4	3.2	2.5	1.5	1.5
	root	1.9	1.3	1.6	1.8	2.4	1.2	1.7	1.4	1.2	1.8
Mn	shoot	*0.3*	1.0	*0.7*	*0.8*	1.0	*0.6*	1.6	*0.6*	*0.5*	*0.7*
	root	*0.5*	*0.6*	1.0	*0.5*	*0.8*	*0.7*	2.4	*0.4*	*0.5*	*0.6*
Pb	shoot	2.5	3.3	3.4	2.7	2.0	2.9	3.5	3.3	10	3.1
	root	14	12	35	14	4.7	16	11	18	19	17
Tl	shoot	8.7	1.5	268	5.9	3.6	8.0	5.2	124	8.8	9.1
	root	418	44	109	59	56	427	77	424	44	540
Zn	shoot	4.0	2.7	8.3	5.9	5.6	10	5.1	5.0	9.3	7.2
	root	14	7.2	18	20	7.9	18	7.9	12	13	16

Ratios <1 are given in italics. *A. collina* (*N* = 7 and 3 for heap and control plants, respectively), *C. hirta* (*N* = 18 and 3), *E. cyparissias* (*N* = 7 and 3), *F. vesca* (*N* = 18 and 2), *H. pilosella* (*N* = 18 and 2), *L. hispidus* (*N* = 7 and 2), *P. arenaria* (*N* = 18 and 1), *P. lanceolata* (*N* = 18 and 3), *R. acetosa* (*N* = 7 and 2), *S. ochroleuca* (*N* = 7 and 3)

Heap plants tended to be impoverished in Ca, Fe, and Mn and enriched in K and Mg when compared to the control plants (Table 2). The concentrations of these elements in tissues were also significantly (*p* < 0.05) affected by the plant species, plant organ (shoot vs. root) and the interaction of both factors. The concentrations of macronutrients, i.e., Ca (Fig. 2e), K (Fig. 2f), and Mg (Fig. 2g), were generally higher in shoots than in roots or similar in the two organs, while the opposite was observed for Fe (Fig. 2h). The accumulation of Mn in plant organs did not follow any consistent pattern (Fig. 2i). The concentrations of Ca, Fe, K, Mg, and Mn varied among sites (Table S1), but the differences were generally smaller in comparison to those for Pb or Tl. The lowest inter-heap variation was observed for macronutrients, whose CVs rarely exceeded 30 %.

### Bioconcentration (BCF) and translocation (TF) factors

Bioconcentration factors (BCF_tot_ and BCF_EDTA_) differed between pollution elements and plant species (Table 3). They were generally <1 for Cd, Pb, and Zn. As expected, the lowest values were found for Pb, indicating very low level of Pb uptake by all species studied. In contrast, BCF_tot_ and BCF_EDTA_ values for Cd and Tl were basically higher and more variable among species; BCF_EDTA_ values for Tl were very high in all cases, suggesting efficient Tl uptake from soil (Table 3). Translocation factors were generally <1 for all pollution elements, which implies their restricted translocation from roots to shoots. TF values varied considerably among species, particularly in the case of Tl and Cd (Table 3).

**Table 3:** Bioconcentration (BCF) and translocation (TF) factors calculated for particular elements and plant species growing on old heaps (means and standard deviations, *N* = 7)

		*A. collina*	*C. hirta*	*E. cyparissias*	*F. vesca*	*H. pilosella*	*L. hispidus*	*P. arenaria*	*P. lanceolata*	*R. acetosa*	*S. ochroleuca*
Ca	BCF_tot_	0.14 (0.12)	0.08 (0.04)	0.14 (0.08)	0.17 (0.07)	0.15 (0.07)	0.17 (0.10)	0.21 (0.15)	0.15 (0.10)	0.17 (0.12)	0.14 (0.11)
	TF	*1.57* (*0.49*)	*2.28* (*0.65*)	*1.60* (*0.34*)	*2.70* (*3.50*)	*1.47* (*0.27*)	0.88 (0.29)	*1.60* (*1.97*)	*1.41* (*0.49*)	0.73 (0.21)	*1.14* (*0.44*)
Cd	BCF_tot_	0.15 (0.05)	0.08 (0.04)	0.24 (0.12)	0.25 (0.09)	0.35 (0.15)	0.05 (0.03)	0.29 (0.11)	0.18 (0.08)	0.04 (0.02)	0.15 (0.10)
	BCF_EDTA_	0.54 (0.19)	0.28 (0.15)	0.87 (0.44)	0.92 (0.38)	*1.27* (*0.61*)	0.17 (0.11)	*1.03* (*0.34*)	0.65 (0.30)	0.15 (0.08)	0.53 (0.33)
	TF	*1.03* (*0.49*)	0.46 (0.20)	0.23 (0.13)	0.29 (0.10)	0.86 (0.23)	0.38 (0.16)	0.30 (0.09)	0.12 (0.06)	0.18 (0.05)	0.07 (0.05)
Fe	BCF_tot_	0.01 (0.00)	0.01 (0.00)	0.01 (0.01)	0.02 (0.01)	0.01 (0.01)	0.01 (0.01)	0.01 (0.01)	0.01 (0.00)	0.01 (0.01)	0.01 (0.00)
	TF	0.65 (0.31)	0.37 (0.20)	0.21 (0.11)	*1.69* (*1.41*)	*1.05* (*0.53*)	0.57 (0.37)	*1.08* (*0.34*)	0.43 (0.22)	0.53 (0.23)	0.49 (0.42)
K	BCF_tot_	*9.10* (*4.86*)	*6.85* (*3.28*)	*7.05* (*3.98*)	*4.18* (*1.57*)	*8.56* (*4.43*)	*8.23* (*5.13*)	*5.11* (*2.82*)	*7.67* (*4.14*)	*6.05* (*2.77*)	*5.97* (*2.64*)
	TF	*1.49* (*0.87*)	*1.80* (*0.89*)	*1.70* (*0.57*)	*2.88* (*0.73*)	*1.14* (*0.31*)	*1.63* (*0.87*)	*2.29* (*0.50*)	*1.76* (*0.91*)	*2.09* (*0.49*)	*1.42* (*0.47*)
Mg	BCF_tot_	0.14 (0.08)	0.10 (0.05)	0.18 (0.09)	0.18 (0.08)	0.18 (0.09)	0.17 (0.08)	0.24 (0.10)	0.17 (0.08)	0.20 (0.10)	0.15 (0.08)
	TF	*1.66* (*0.32*)	*1.38* (*0.25*)	*2.20* (*0.39*)	*1.85* (*0.41*)	*1.57* (*0.43*)	*1.81* (*0.37*)	*1.36* (*0.16*)	*1.43* (*0.36*)	*2.23* (*0.69*)	*1.14* (*0.35*)
Mn	BCF_tot_	0.03 (0.02)	0.06 (0.02)	0.05 (0.02)	0.07 (0.07)	0.04 (0.02)	0.04 (0.01)	0.06 (0.02)	0.03 (0.01)	0.03 (0.02)	0.03 (0.02)
	TF	*1.07* (*0.65*)	*2.83* (*1.03*)	0.80 (0.36)	*1.38* (*0.69*)	*1.50* (*0.61*)	0.61 (0.19)	0.91 (0.37)	0.45 (0.20)	0.61 (0.22)	0.44 (0.20)
Pb	BCF_tot_	0.02 (0.00)	0.01 (0.00)	0.03 (0.01)	0.04 (0.02)	0.02 (0.01)	0.02 (0.01)	0.03 (0.01)	0.02 (0.01)	0.02 (0.02)	0.02 (0.01)
	BCF_EDTA_	0.07 (0.03)	0.06 (0.02)	0.12 (0.05)	0.18 (0.09)	0.09 (0.05)	0.09 (0.04)	0.13 (0.08)	0.07 (0.03)	0.09 (0.06)	0.08 (0.04)
	TF	0.15 (0.06)	0.20 (0.15)	0.13 (0.10)	0.13 (0.09)	0.41 (0.23)	0.30 (0.23)	0.24 (0.10)	0.20 (0.18)	0.20 (0.13)	0.21 (0.19)
Tl	BCF_tot_	0.37 (0.44)	0.07 (0.15)	0.53 (0.80)	0.11 (0.11)	0.25 (0.32)	0.39 (0.52)	0.24 (0.38)	*1.52* (*1.77*)	0.07 (0.12)	0.42 (0.42)
	BCF_EDTA_	*6.40* (*6.20*)	*1.15* (*2.26*)	*9.11* (*12.8*)	*2.08* (*2.14*)	*4.46* (*4.70*)	*6.69* (*7.49*)	*4.12* (*5.48*)	*27.1* (*25.5*)	*1.20* (*1.93*)	*7.85* (*7.32*)
	TF	0.03 (0.03)	0.90 (1.55)	*2.77* (*3.11*)	0.38 (0.51)	0.21 (0.33)	0.06 (0.10)	0.18 (0.22)	0.36 (0.20)	0.60 (0.19)	0.09 (0.18)
Zn	BCF_tot_	0.02 (0.01)	0.02 (0.01)	0.05 (0.02)	0.05 (0.02)	0.02 (0.01)	0.03 (0.02)	0.06 (0.03)	0.04 (0.01)	0.02 (0.02)	0.03 (0.01)
	BCF_EDTA_	0.24 (0.10)	0.22 (0.09)	0.62 (0.21)	0.68 (0.39)	0.34 (0.14)	0.45 (0.14)	0.78 (0.30)	0.53 (0.16)	0.32 (0.16)	0.41 (0.21)
	TF	0.72 (0.45)	0.42 (0.14)	0.43 (0.21)	0.15 (0.03)	0.90 (0.29)	0.67 (0.20)	0.22 (0.06)	0.23 (0.07)	0.52 (0.26)	0.26 (0.11)

BCFs and TFs >1 are presented in *italics*

BCF_tot_ values for Ca, Mg, and, particularly, Fe, and Mn were low, contrasting with those for K; the concentrations of K in plant tissues were a few times higher than those in soil (Table 3). Ca, K, and Mg were easily transported to shoots, as indicated by TF values >1. TF values for Fe and Mn were <1 in most species; however, the reverse situation was quite frequent (Table 3).

### Concentrations of elements in plants in relation to soil

The concentrations of Cd, Pb, and Zn in roots and shoots of nearly all species increased significantly with increasing metal concentration in soil (Table 4). The increase of Cd, Pb, and Zn in plant tissues was more pronounced for roots than shoots in most species (Fig. S1a for *P. arenaria*), but there were exceptions, in which the increase was similar in the above- and belowground organs (Fig. S1b for *H. pilosella*). The concentrations of Cd, Pb, and Zn in plant tissues generally reflected well the corresponding concentrations (both total and available, i.e., BaCl_2_-, EDTA-, and water-extractable) of metals in the soil (Table 4). These relationships were quite strong; correlation coefficients (r) often exceeded 0.7 or even 0.8. The relationships between the Pb content in the soil and plants were generally stronger for roots than for shoots, whereas patterns for Cd and Zn were more variable and depended on the plant species (Table 4). Soil-plant relationships for Tl were less consistent (Table 4). Total Mg was positively correlated to the Mg content in the shoots of *C. hirta* (*r* = 0.69), *F. vesca* (*r* = 0.82) and *P. arenaria* (*r* = 0.64), and in the roots of *F. vesca* (*r* = 0.62), *P. arenaria* (*r* = 0.56), and *P. lanceolata* (*r* = 0.48), while BaCl_2_-extractable Mg was correlated with the Mg content in the shoots (*r* = 0.57) and roots of *F. vesca* (*r* = 0.51). Significant soil-plant correlations for other elements, i.e., Ca, Fe, K, and Mn, were sporadic or non-existent.

**Table 4:** Correlations between heavy metals in soil and plant tissues (*N* = 18)

		*C. hirta*		*F. vesca*		*H. pilosella*		*P. arenaria*		*P. lanceolata*	
		shoot	root	shoot	root	shoot	root	shoot	root	shoot	root
Cd	Total	0.51*	0.67**	0.87***	0.82***	0.76***	0.79***	0.89***	0.89***	0.53*	0.84***
	EDTA-extractable	0.59*	0.67**	0.78***	0.74***	0.73***	0.74***	0.86***	0.86***	0.61**	0.87***
	BaCl_2_-extractable	0.53*	0.73***	0.85***	0.77***	0.81***	0.83***	0.82***	0.79***	0.57*	0.83***
	H_2_O-extractable	0.64**	0.71**	0.68**	0.68**	0.72***	0.71**	0.67**	0.71***	0.72***	0.74***
Pb	Total	0.57*	0.85***	0.63**	0.86***	0.67**	0.74***	0.55*	0.65**	0.58*	0.80***
	EDTA-extractable	0.59**	0.86***	0.64**	0.85***	0.63**	0.68**	0.59*	0.64**	0.66**	0.86***
	H_2_O-extractable	0.59*	0.79***	0.56*	0.67**	ns	0.55*	ns	ns	ns	0.70**
Tl	Total	ns	ns	ns	ns	ns	ns	ns	ns	ns	ns
	EDTA-extractable	ns	ns	0.52*	ns	ns	ns	ns	ns	ns	ns
	H_2_O-extractable	0.66**	0.49*	0.66**	ns	0.57*	ns	0.55*	ns	ns	ns
Zn	Total	ns	0.69**	0.65**	0.75***	0.74***	0.68**	0.72***	0.82***	0.66**	0.75***
	EDTA-extractable	ns	0.68**	0.63**	0.68**	0.78***	0.61**	0.79***	0.82***	0.75***	0.85***
	BaCl_2_-extractable	ns	0.74***	0.58*	0.66**	0.64**	0.53*	0.73***	0.78***	0.67**	0.78***
	H_2_O-extractable	ns	0.59**	0.53*	0.70**	0.70**	0.64**	ns	0.60**	0.60**	0.63**

***p < 0.001, **p < 0.01, *p < 0.05

*ns* not significant

## Discussion

The concentrations of Cd, Pb, Tl, and Zn were elevated in the soil from old heaps, reaching sometimes extreme levels. These concentrations were much higher than those reported typically for unpolluted soils, while they were similar to the values measured at other sites affected by metal ore mining and processing (Kabata-Pendias 2011). Soils that had developed on old heaps were also rich in total Fe and Mn in comparison to control soils, which results from the presence of Fe sulfides or sulfates as well as Mn and Fe oxides in Zn-Pb ores (Cabala et al. 2009). According to Kabata-Pendias (2011). similar or even higher amounts of Mn are found in some non-metalliferous (calcareous) soils. The heap soils were also characterized by high concentrations of Mg and Ca, which originated from the weathering of gangue carbonates—dolomite and calcite (Cabala et al. 2009; Stefanowicz et al. 2014).

The concentrations of the main pollutants, i.e., Cd, Pb, Tl, and Zn, in the tissues of plants growing on heaps were much higher than values for the “reference plant” introduced by Markert (1992). i.e., 0.05 mg Cd kg^-1^, 1 mg Pb kg^-1^, 0.05 mg Tl kg^-1^, and 50 mg Zn kg^-1^. Heap plants contained much more Cd, Pb, Tl, and Zn than controls, reaching levels typical for plants from the areas of metal mining and processing (Godzik 1993; Kabata-Pendias 2011; Xiao et al. 2004). As expected, metal concentrations differed between the plant species and plant organs. The highest concentrations of Cd, Pb, and Zn were detected in the roots of *E. cyparissias*, *F. vesca*, *P. arenaria*, *P. lanceolata*, and *S. ochroleuca*, whereas Tl in the roots of *P. lanceolata* and *S. ochroleuca*. Of these plants, *P. lanceolata* has often been tested for its ability to accumulate heavy metals: the tissue concentrations of Cd, Pb, Tl, and Zn in this study were largely similar to the concentrations reported by Abe et al. (2008). Álvarez-Ayuso et al. (2013). Nadgórska-Socha et al. (2013). Orłowska et al. (2002) and Wójcik et al. (2014). but markedly lower than those reported by Wierzbicka et al. (2004) or Dimitrova and Yurukova (2005). Concentrations of Cd, Pb, or Zn in the shoots of *C. hirta*, *H. pilosella*, *L. hispidus*, *P. arenaria*, *R. acetosa*, and *S. ochroleuca* growing on old heaps were consistent with the concentrations measured by Turnau et al. (2010) and Wójcik et al. (2014) in plants from metal-polluted areas.

Average Fe concentrations in both heap and control plants oscillated around the reference value (150 mg kg^-1^; Markert 1992) and generally fell within the ranges found naturally in plants (Kabata-Pendias 2011). Fe concentrations in the heap plants were broadly similar to those given by Godzik (1993). but several times lower than some of the values reported by other authors. For example, *H. pilosella* growing on tailings accumulated on average 1500 mg Fe kg^-1^ in the leaves (Turnau et al. 2010). *P. lanceolata* from calamine spoils over 1000 mg kg^-1^ in roots (Szarek-Łukaszewska and Niklińska 2002), and *F. vesca* from slags up to 1700 mg kg^-1^ (Antosiewicz et al. 2008).

The concentrations of Mn were low in the old heap and control plants in comparison to the “reference plant” (200 mg kg^-1^; Markert 1992). The lowest average Mn concentrations were detected in *P. lanceolata*, *S. ochroleuca*, and *R. acetosa*; values for these species were lower than 15–25 mg Mn kg^-1^, which is considered as the critical deficiency level for most plants (Kabata-Pendias 2011). Moreover, the Fe/Mn ratio was >2.5 for 80 % of the plant shoot samples from the heap and control sites (data not shown), reaching in many cases very high values; this also indicated Mn deficiency (Kabata-Pendias 2011). Mn and Fe are interrelated in metabolic functions and the Fe/Mn tissue ratio between 1.5 and 2.5 is necessary for a plant to be healthy (Kabata-Pendias 2011). High Fe/Mn tissue ratios detected in this study suggests that the Fe deficiency could be expected neither in control nor in the heap plants, although Fe, like Mn, is poorly available in the well-oxidized neutral and alkaline soils (Lambers et al. 2008; Kabata-Pendias 2011).

The concentrations of Ca and Mg in plant tissues from the heap and control sites generally fell within the range typical for plants not exposed to stress, i.e., 1–44 g Ca kg^-1^ and 1–4 g Mg kg^-1^ (Bose et al. 2011; Broadley et al. 2003) and varied around the reference values, i.e., 10 g Ca kg^-1^ and 2 g Mg kg^-1^ (Markert 1992). The concentrations of K in both heap and control plants were below or near the lower limit of the normal range observed in plants, i.e., 10–100 g kg^-1^ (Britto and Kronzucker 2008) and were generally lower than the K concentration of the “reference plant” (19 g kg^-1^; Markert 1992). According to expectations, the concentrations of Ca, K, and Mg seemed to differ between the heap and control plants. Ca, Fe, and Mn tended to be lower, while K and Mg were higher in the heap than in control plants. These findings are somewhat surprising, as the concentrations of available (BaCl_2_-extractable) forms of Ca, Fe, K, and Mn were either higher in the heap than in control or similar in the two soils. Our results are in contrast with Turnau et al. (2010) who found that many plant species growing on post-flotation wastes contained more Fe and Ca in the leaves than control plants, although the availability of these elements in the waste was low. The opposite was observed for K—its concentrations were generally lower in plants from tailings, but this probably resulted from a lower availability of this element at that site (Turnau et al. 2010). Evident enrichment of heap plant tissues in Mg detected in our study resulted probably from the higher Mg concentration and availability in heap soils, originating from the high dolomite content in the waste. Godzik (1993) often reported more Fe and Mg in the heap plants, but the accumulation of these elements and Ca in the heap and control plants depended on the species and organ. Patterns of element accumulation in plants depend on the availability of a given element in the soil, plant physiology, and complex interactions between elements, particularly in the heavy-metal rich environments. Heavy metals may cause disturbances in the K/Ca ratio in plant tissues, followed by alterations in the water balance, and Fe deficiency due to decreased uptake or immobilization in the roots (Siedlecka 1995). Cd may replace Mn during the uptake process, whereas the Fe-Ca interaction suppresses Fe availability (Kabata-Pendias 2011). Goss and Carvalho (1992) reported that the maximum Mn concentration in shoots fell as the concentration of Mg in the nutrient solution increased; Mg also reduced the quantity of Mn translocated from the roots. Changes in the tissue concentrations of some nutrients in plants growing in heavy metal-polluted soil may result from the competition for uptake between cations that have similar ionic radii (Siedlecka 1995). Antosiewicz (2005) claimed that the inhibition of Ca channels in a cell may result either from the competitive transport or from channel blockage by Pb. The uptake of Ca, Fe, or Mn by roots may be decreased if the roots are damaged or their growth is inhibited by heavy metals (Godbold and Kettner 1991; Ouzounidou et al. 1995; Siedlecka 1995). Heavy metals may cause root membrane lipid peroxidation, damaging the permeability barrier of root cells, influence the activity of ATP-ases and other carriers, as well as decrease root respiration, which may reduce the uptake of elements actively transported into the roots (De Vos et al. 1991; Ros et al. 1992; Siedlecka 1995).

The efficiency of element acquisition and accumulation in plant tissues differed considerably between the elements, plant species, and organs (shoots vs. roots). K was taken up from the soil quite efficiently and all macronutrients were effectively translocated to shoots. In contrast, Cd, Pb, Tl, and Zn were accumulated mainly in roots; the restricted translocation to the shoots was most noticeable for Pb, which is a well-known phenomenon (Kabata-Pendias 2011; Siedlecka 1995). Plant species that have evolved both tolerant and non-tolerant ecotypes, such as species tested in this study, often behave as excluders – plants that restrict heavy metal transport from roots to shoots (Baker 1981; Pollard et al. 2002).

Inter-heap variability in metal accumulation of individual species was high, which might be the result of both soil- and population-specific properties (Kabata-Pendias 2011; Pošćić et al. 2013). An interesting observation was made in this study for *E. cyparissias*; its populations largely differed in the Tl root-shoot translocation. The concentration of H_2_O-extractable Tl explained well the variation in Tl concentration in the shoots and roots of *E. cyparissias*, but not the variation in TF (data not shown). Tl translocation may be population-specific, as different populations may have different Tl accumulation strategies. It has been already shown for *Biscutella laevigata* that the ability to hyperaccumulate Tl is not a species-wide property (Pošćić et al. 2013). More work is needed, including extensive sampling of *E. cyparissias* populations in the field and their cultivation under controlled conditions, to elucidate the differences in the Tl translocation in this plant species.

The concentrations of Cd, Pb, and Zn in plant tissues were mainly positively related to their concentrations in the soil. These findings are in agreement with observations described by other authors, albeit soil-plant relationships may depend on a metal itself, metal extraction from the soil, soil type, or plant species (Chojnacka et al. 2005; Deng et al. 2004; Kabata-Pendias 2011). Deng et al. (2004) claimed that metals in the underground tissues generally had stronger positive correlations with metals in the substratum than the aboveground tissues. It was also often observed in our study, being particularly pronounced for Pb. Significant soil-plant correlations for Ca, Fe, K, Mg, Mn, and Tl were less frequently observed. This may be explained in part by the inter-heap variation in the concentrations of soil elements, which was generally high for all forms of Cd, Pb and Zn, and low for other elements. Large variation in metal concentrations facilitates the detection of soil-plant relationships.

## Conclusions

Old heaps left by historical mining for Zn-Pb ores are “hot spots” of heavy metal contamination, and thus affect neighboring agricultural land and possibly pose a threat to the environment and people (Stefanowicz et al. 2014). This study proved that plants overgrowing old heaps accumulate much higher amounts of Cd, Pb, Tl, and Zn than the plants from control sites. The highest contents of heavy metals were found in the roots, but their levels in the shoots were elevated as well, implying a risk of transport of toxicants along the food chain. Considering the fact that old heaps frequently occur near human settlements, arable fields, and vegetable gardens, and that the dolomite waste rock, which forms the heaps, is sometimes collected and re-used by local residents for different purposes, e.g., to level the ground around their homes, small-scale crop production on these vast post-mining areas may be hazardous (Stefanowicz et al. 2014).

From among the ten plant species tested in our study, *F. vesca*, *P. arenaria*, *P. lanceolata*, and *S. ochroleuca* seem to be the most suitable for reclamation of metal-polluted areas. They accumulate high amounts of heavy metals in the roots and have a relatively high (although basically <1) plant/soil ratio as well as relatively low shoot/root ratio, so they can be useful in phytostabilization. These species are components of naturally valuable xerothermic grasslands that develop spontaneously in metal-polluted areas and may be potentially used in directed succession.

## Electronic supplementary material

Below is the link to the electronic supplementary material.

Fig. S1 Relationships between Cd concentrations in the soil and tissues of *P. arenaria* (**a**) and *H. pilosella* (**b**). The graph is based on non-transformed data. (EPS 468 kb)

Table S1 (DOCX 38 kb)
