# Supplementary material for: The accumulation of elements in plants growing spontaneously on small heaps left by the historical Zn-Pb ore mining
Source: Environ Sci Pollut Res Int. 2015 Dec 4;23:6524–34. doi: 10.1007/s11356-015-5859-7 (PMC4820495; doi:10.1007/s11356-015-5859-7)
Supplement: Supplementary file 2 — (DOCX 38 kb) [file 11356_2015_5859_MOESM2_ESM.docx]

Table S1. Ranges of metal concentrations in plants from heaps and control sites.

|  | | | *Achillea*  *collina* | *Carex*  *hirta* | *Euphorbia*  *cyparissias* | *Fragaria*  *vesca* | *Hieracium*  *pilosella* | *Leontodon*  *hispidus* | *Potentilla*  *arenaria* | *Plantago*  *lanceolata* | *Rumex*  *acetosa* | *Scabiosa*  *ochroleuca* |
| --- | --- | --- | --- | --- | --- | --- | --- | --- | --- | --- | --- | --- |
| Ca (g kg^-1^) | heap | shoot | 5–9 | 3–8 | 7–11 | 2–14 | 4–11 | 5–12 | 4–18 | 4–10 | 4–9 | 5–9 |
|  |  | root | 3–6 | 1–3 | 4–10 | 1–15 | 3–8 | 7–11 | 1–14 | 4–10 | 7–13 | 4–9 |
|  | control | shoot | 5–13 | 5–8 | 8–10 | 6–7 | 6–13 | 9–10 | 7 | 5–6 | 6–8 | 5–7 |
|  |  | root | 9–10 | 2–6 | 1–11 | 2–29 | 9–17 | 12 | 1 | 1–11 | 1–2 | 1–19 |
| Cd (mg kg^-1^) | heap | shoot | 8–18 | 1–12 | 5–11 | 2–14 | 4–81 | 1–6 | 1–30 | 1–6 | 1–4 | 1–2 |
|  |  | root | 6–23 | 1–19 | 14–92 | 4–75 | 5–70 | 2–26 | 3–127 | 3–70 | 3–20 | 8–106 |
|  | control | shoot | 0.3–0.8 | 0.2–1.6 | 0.4–0.7 | 0.6–0.8 | 0.5–0.8 | 0.7 | 0.9 | 0.3–0.7 | 0.5–0.6 | 0.4–0.5 |
|  |  | root | 0.6–0.8 | 0.3–1.6 | 0.4–0.6 | 0.7–1.2 | 0.6–1.0 | 0.5–0.6 | 0.7 | 0.6–0.8 | 1.1–1.2 | 0.6–0.8 |
| Fe (mg kg^-1^) | heap | shoot | 62–152 | 78–189 | 40–97 | 188–532 | 103–425 | 75–353 | 185–1215 | 34–197 | 44–231 | 47–132 |
|  |  | root | 69–197 | 143–509 | 122–739 | 97–996 | 86–771 | 118–762 | 101–1110 | 91–1608 | 82–534 | 86–695 |
|  | control | shoot | 116–216 | 122–144 | 92–238 | 143–183 | 163–305 | 160–224 | 192 | 38–263 | 48–180 | 59–150 |
|  |  | root | 226–678 | 205–672 | 224–838 | 426–906 | 348–565 | 418–820 | 480 | 305–713 | 165–349 | 219–574 |
| K (g kg^-1^) | heap | shoot | 9–24 | 7–17 | 8–15 | 5–15 | 8–24 | 9–24 | 5–15 | 6–19 | 7–15 | 7–11 |
|  |  | root | 7–17 | 5–9 | 6–9 | 2–5 | 7–17 | 6–11 | 2–5 | 6–17 | 4–8 | 5–10 |
|  | control | shoot | 8–13 | 7–9 | 7–9 | 3–7 | 9–11 | 10 | 8 | 7–8 | 7–11 | 7–8 |
|  |  | root | 10–16 | 6–9 | 6–7 | 3 | 10–14 | 8–11 | 4 | 6–9 | 5 | 7–9 |
| Mg (g kg^-1^) | heap | shoot | 2–3 | 1–4 | 3–6 | 2–6 | 2–5 | 3–6 | 2–9 | 2–7 | 3–8 | 2–4 |
|  |  | root | 1–2 | 1–2 | 1–2 | 1–3 | 1–4 | 1–3 | 2–4 | 1–3 | 1–2 | 2–3 |
|  | control | shoot | 1–2 | 1–2 | 2 | 1–2 | 1–2 | 3 | 2 | 1–2 | 3 | 1–2 |
|  |  | root | 0.5–1 | 1 | 1 | 1 | 1 | 1–2 | 2 | 1–3 | 2 | 1 |
| Mn (mg kg^-1^) | heap | shoot | 15–29 | 21–123 | 21–42 | 19–108 | 19–113 | 10–35 | 15–122 | 7–44 | 6–26 | 7–15 |
|  |  | root | 12–35 | 13–38 | 19–73 | 10–94 | 11–97 | 16–61 | 24–133 | 15–98 | 10–57 | 15–49 |
|  | control | shoot | 44–73 | 41–91 | 21–44 | 47–82 | 34–81 | 21–49 | 35 | 6–36 | 20–38 | 8–28 |
|  |  | root | 21–66 | 34–47 | 26–61 | 50–134 | 27–94 | 20–83 | 25 | 15–267 | 37–65 | 17–102 |
| Pb (mg kg^-1^) | heap | shoot | 4–45 | 3–27 | 4–64 | 8–34 | 3–46 | 9–34 | 7–64 | 2–22 | 4–77 | 5–44 |
|  |  | root | 18–264 | 7–272 | 23–485 | 20–693 | 11–266 | 14–314 | 12–554 | 8–527 | 14–611 | 14–531 |
|  | control | shoot | 4–7 | 1–3 | 3–6 | 6 | 5–12 | 6–7 | 7 | 1–4 | 2 | 3–7 |
|  |  | root | 6–7 | 4–5 | 5 | 8–13 | 9–13 | 7–8 | 10 | 5–6 | 8–11 | 7–8 |
| Tl (µg kg^-1^) | heap | shoot | 25–1380 | 21–250 | 36–43580 | 32–1320 | 27–1080 | 27–1190 | 28–1650 | 35–20940 | 27–1430 | 30–1870 |
|  |  | root | 2403–17656 | 8–5753 | 47–7422 | 26–7534 | 27–13812 | 96–19924 | 27–14266 | 43–54593 | 40–6116 | 67–38949 |
|  | control | shoot | 36–39 | 29–39 | 36–41 | 31–43 | 36–42 | 40–47 | 34 | 32–45 | 48 | 37–48 |
|  |  | root | 16–20 | 8–21 | 14–22 | 16–39 | 28–81 | 12–23 | 35 | 21–32 | 26–31 | 20 |
| Zn (mg kg^-1^) | heap | shoot | 66–452 | 76–342 | 232–766 | 50–451 | 97–565 | 205–678 | 68–688 | 69–692 | 65–719 | 89–318 |
|  |  | root | 100–526 | 60–513 | 541–1630 | 193–2068 | 40–805 | 242–1026 | 175–2657 | 160–2616 | 231–880 | 342–1221 |
|  | control | shoot | 18–71 | 32–67 | 34–65 | 18–37 | 32–68 | 28–48 | 51 | 23–75 | 11–47 | 18–29 |
|  |  | root | 15–28 | 28–51 | 26–92 | 30–63 | 24–56 | 28–40 | 124 | 63–83 | 18–54 | 41–50 |

Heaps: N=18 for *C. hirta, F. vesca, H. pilosella, P. arenaria* and *P. lanceolata*, N=7 for other species. Controls: N=1 for *P. arenaria*, N=2 for *F. vesca, H. pilosella, L. hispidus* and *R. acetosa*, N=3 for other species
